# Alterations of Extracellular Matrix Components in the Course of Juvenile Idiopathic Arthritis

**DOI:** 10.3390/metabo11030132

**Published:** 2021-02-25

**Authors:** Magdalena Wojdas, Klaudia Dąbkowska, Katarzyna Winsz-Szczotka

**Affiliations:** Department of Clinical Chemistry and Laboratory Diagnostics, Faculty of Pharmaceutical Sciences in Sosnowiec, Medical University of Silesia, ul. Jedności 8, 41-200 Sosnowiec, Poland; klaudia_092@vp.pl (K.D.); winsz@sum.edu.pl (K.W.-S.)

**Keywords:** juvenile idiopathic arthritis, extracellular matrix, proteoglycans, matrix metalloproteinases, reactive oxygen species

## Abstract

Juvenile idiopathic arthritis (JIA) is the most common group of chronic connective tissue diseases in children that is accompanied by joint structure and function disorders. Inflammation underlying the pathogenic changes in JIA, caused by hypersecretion of proinflammatory cytokines, leads to the destruction of articular cartilage. The degradation which progresses with the duration of JIA is not compensated by the extent of repair processes. These disorders are attributed in particular to changes in homeostasis of extracellular matrix (ECM) components, including proteoglycans, that forms articular cartilage. Changes in metabolism of matrix components, associated with the disturbance of their degradation and biosynthesis processes, are the basis of the progressive wear of joint structures observed in the course of JIA. Clinical evaluation and radiographic imaging are current methods to identify the destruction. The aim of this paper is to review enzymatic and non-enzymatic factors involved in catabolism of matrix components and molecules stimulating their biosynthesis. Therefore, we discuss the changes in these factors in body fluids of children with JIA and their potential diagnostic use in the assessment of disease activity. Understanding the changes in ECM components in the course of the child-hood arthritis may provide the introduction of both new diagnostic tools and new therapeutic strategies in children with JIA.

## 1. Introduction

Juvenile idiopathic arthritis (JIA) is the most common group of chronic connective tissue diseases in children that is accompanied by joint structure and function disorders. Clinical symptoms indicating pathological inflammatory processes in the joints, i.e., pain, presence of exudate or limitation of mobility, which allow the diagnosis of JIA, must be present in the patient for at least six weeks. The diagnosis of JIA, due to its complex etiopathogenesis, heterogeneity of clinical manifestations, and lack of pathognomonic symptoms, is a complex process and is based on the collection of a detailed history from the patient and family, a physical examination of the patient, and the performance of diagnostic laboratory tests and imaging studies [1,2]. The heterogeneous clinical expression of the disease has become the basis for recognition by the International League of Associations for Rheumatology (ILAR) six subtypes of JIA: Systemic JIA, oligoarticular JIA (including a persistent and expanding form), polyarticular JIA (rheumatoid factor (RF)-negative and RF-positive form), enthesitis-related arthritis, psoriatic arthritis and undifferentiated JIA [3,4,5]. Scientists are working on defining new JIA classification criteria and different forms of the disease [3,6]. Arthropathy develops in children with genetically determined disorders of the immune response, more often in people exposed to external factors such as stress, bacterial infections (i.e., Mycoplasma pneumoniae, Borrelia burgdorferi, Yersinia enterocolitica, Proteus mirabilis or viral infections), parvovirus B19, rubella virus, influenza virus, cytomegalovirus, Epstein-Barr virus [7,8,9,10]. The infectious factors, by interfering with the metabolism of the immune system, lead to the synthesis of autoantibodies as well as changes in the synthesis of signaling molecules and adhesion molecules. As a result, inflammation develops within the joint structures, the formation of which is associated with the activation of numerous pro-inflammatory cytokines, including tumor necrosis factor α (TNF-α) and interleukin (IL) i.e.,—IL-1, IL-6, IL-8, IL -12, IL-15, IL-17, IL-18 [11,12,13,14]. Pro-inflammatory cytokines lead to the destruction of articular cartilage, which progresses with the duration of JIA, not compensated by the extent of repair processes [15,16,17]. These disorders are attributed in particular to changes in homeostasis of extracellular matrix components of the connective tissue that forms articular cartilage. Extracellular matrix (ECM) is a multi-component, organized structure that fills the spaces between chondrocytes. The cartilage ECM consists mainly of collagen proteins, which account for about two-thirds of the dry weight of adult articular cartilage. Type II collagen represents 90% to 95% of the collagen in ECM, while type VI, IX, X, XI, XII, XIV are found in smaller amounts. The minor collagens help to form and stabilize the type II collagen fibril network [18]. Collagen fibrils provide cartilage with tensile strength, which depends on the extensive cross-linking of the collagen. Proteolytic and mechanical damage to the fibrillar network is believed to be a key, perhaps irreversible, stage in the destruction of joint cartilages in arthritis [19]. In addition, the cartilage matrix—in about one-thirds of the dry weight—is formed by proteoglycan (PG) aggregates, including mainly aggrecan and small amounts of decorin, biglycan, fibromodulin, lumican or proteoglycan-100. In the structure of the matrix small amounts of non-collagen proteins are found, including fibronectin, tenascin, chondronectin, vitronectin, thrombospondin and matrilin [20,21,22,23]. PGs play a special role in maintaining the mechanical-immunological properties of cartilage. PGs are co-formed by the core protein to which heteropolysaccharide chains of glycosaminoglycans (GAGs) are attached, i.e., chondroitin sulfates, keratan sulfates, dermatan sulfates and trace amounts of heparanosulphate glycosaminoglycans. The cartilage strength and load resistance of PGs result in particular from the ability of the aggrecan to be aggregated with the GAG chain, i.e., hyaluronic acid (HA) [24,25,26]. HA has an important role in joint biomechanics, where it is partially responsible for lubrication and viscoelasticity of the synovial fluid. HA is involved in many cellular interactions, i.e., cell differentiation, proliferation and development [27]. In the course of JIA, changes in HA concentrations in body fluids were found. It is suggested that that serum HA measurement is useful for diagnosing systemic and polyarticular JIA [28].

To maintain healthy cartilage tissue, the ECM must regenerate by normal remodeling, mediated by the cells that form the matrix, in which old or damaged compounds are broken down in a specific sequence of degradation process and replaced by new ingredients. However, during pathological conditions, such as inflammation, the damage-repair balance is disturbed [29]. The changes in metabolism of matrix components, associated with the disturbance of their degradation and biosynthesis processes, are the basis of the progressive wear of joint structures observed in the course of JIA. Catabolic processes of ECM components take place with specific proteolytic enzymes and non-specific reactive oxygen species (ROS) and reactive nitrogen species (RNS), which stimulate extracellular degradation of PGs. The main enzymes involved in the process of PGs protein core digestion are matrix metalloproteinases (MMPs) and ADAMTS (A Disintegrin and Metalloproteinase with Thrombospondin motifs) proteins [30,31,32,33].

## 2. Matrix Metalloproteinases

MMPs belong to the group of multidomain zinc endopeptidases involved in the digestion of ECM components. MMPs are able to degrade the structures on the surface of cells, i.e., adhesion proteins, apoptosis mediators, receptors, chemokines, cytokines, growth factors and pro-MMPs, thus regulating their activity [34,35]. MMPs participate in many physiological processes such as embryogenesis, angiogenesis, apoptosis and changes in their activity are the basis for the development of diseases such as cancers, autoimmune, inflammatory and degenerative diseases [36,37]. MMPs are characterized by a wide variety of structure, substrate specificity and mechanism of action. It was the basis for distinguishing several subclasses of these enzymes in the Table 1. MMPs are synthesized in the form of inactive precursors—zymogens—by many cell types, including fibroblasts, osteoblasts, mast cells, keratinocytes, endothelial cells and inflammatory cells such as macrophages, lymphocytes, neutrophils and eosinophils [38,39,40]. The process of zymogen activation, which involves changing the conformation of the MMPs molecule, occurs with the participation of proteases such as trypsin, chymotrypsin, elastase, plasmin and other MMPs. It may also occur with the participation of non-specific factors, such as oxidized glutathione, ROS, low pH or elevated temperature [33]. The activity of these enzymes is strictly controlled by numerous macromolecular interactions, which directs MMPs to specific areas in the extracellular space, thus accumulating near the target substrates. Collagen, laminine, fibronectin, and GAGs are among the matrix components that bind MMPs. For example, glycan chains of chondroitin sulfates, binding MMP3, contribute to tissue degradation of PGs co-created by these GAGs [41,42]. To maintain a balance that prevents excessive enzymatic degradation of ECM components, tissue inhibitors of metalloproteinases (TIMPs) and a number of nonspecific protease inhibitors are produced e.g., α2- macroglobulin, corticosteroids, retinoic acid, thyroid hormones, sex hormones, heparin or IL-4 [43,44,45,46].

Proteolytic-antiproteolytic imbalances observed in children with JIA, reflected in changes in MMPs concentrations and their tissue inhibitors in body fluids, contribute to disturbances in the metabolism of ECM components of cartilage tissue. Among MMPs, the key role in ECM remodeling associated with JIA is assigned to MMP-3. This enzyme is capable of degradation of most matrix components, as well as activating other MMPs, which enhances proteolytic catabolic processes [49,50]. The study concerning the assessment of MMPs in body fluids, i.e., in blood and synovial fluid (SF), confirm that children with JIA have high MMP-3 levels in comparison to healthy children regardless of the type of arthropathy, age and duration of disease [51]. The subsequent studies indicated that MMP-3 concentration correlates with platelet count, erythrocyte sedimentation rate and C-reactive protein concentration in children which are routinely determined indicators of inflammation [52,53]. The above has led to the hypothesis that MMP-3 could be used in the evaluation of disease activity and progression. Conventional treatment with methotrexate, oral corticosteroids and non-steroidal anti-inflammatory drugs, leading to clinical improvement in patients, does not contribute to the normalization of MMP-3 in the blood [54]. A simultaneous analysis of the results of MMP-3 concentration and its tissue inhibitor shows that the increase in TIMP-1 concentration that accompanies the JIA does not compensate for the proteolytic activity of the mentioned MMP [53]. Analogous results of these parameters are found in the group of adults with diagnosed rheumatoid arthritis (RA) [55,56]. This indicates that similar factors contribute to the etiopathogenesis of both diseases. Apart from MMP-3 concentration changes, the participation of other MMPs in the development of JIA is also indicated. Thus, Peake et al. [57] reported an increased activity of MMP-2 and MMP-9 in SF and blood serum of patients using quantitative protein substrate zymography method to evaluate MMPs concentration. The authors suggest that MMPs from the gelatinase group may be markers of joint destruction in the active phase of JIA. The results of Kobus et al. [58], concerning the analysis of MMPs concentrations in saliva of patients with JIA, confirmed a statistically significantly higher concentration of MMP-9. MMP-9 and TIMP-1 have a positive correlation with the occurrence of gingivitis in the group of patients. It is also found that the level of MMP-2, MMP-8 and TIMP-1 in saliva of children with JIA is comparable to the level in the control group [58]. These results are slightly different from those obtained by Brik et al. [59]. The findings of authors showed a decrease in the level of MMP-2, MMP-3 and MMP-9 in saliva of children with diagnosed arthropathy. The authors suggest that there is a local mechanism, associated with the influence of antioxidants, that determines the reduction of the concentrations of these enzymes in saliva of sick children. Increasing the content of antioxidants in saliva most probably reduces the formation of MMPs in salivary glands. Differences in blood MMP concentrations compared to saliva seem to indicate that saliva, despite its analytical advantages, should not be used as a material to evaluate proteolytic-antiproteolytic balance and to assess the efficacy of treatment in children with JIA [59].

To determine the effect of proteolytic enzymes on the pathogenesis of JIA, it is important to assess the amount of TIMPs in the body fluids of children with JIA. The analysis of TIMP-1 concentrations indicates an increase in its level both in serum and SF in patients [54,60]. Serum concentration is lower in relation to concentration obtained in the SF, which confirms the thesis that the inflammatory process in the joints contributes to local proteolytic-antiproteolytic balance disorders [60]. TIMP-1 and TIMP-2 analysis in adult patients with inflammatory joint diseases show comparable alterations to children [61]. Comparison of tissue inhibitor concentrations in children with different JIA subtypes seems to be difficult due to differences in the obtained results. Peake et al. [52] proved no significant differences between JIA subtypes for TIMP-1 levels in SF or serum. Agarwal et al. [62] indicated that patients with enthesitis-related arthritis have higher SF levels of the inhibitor in comparison to children with polyarticular JIA and adult with RA. This indicates the complexity of the mechanisms accompanying enzymatic disorders that determine the occurrence and progression of arthritis.

## 3. ADAM and ADAMTS

ADAM (a disintegrin-like and metalloproteinase) belong to the group of transmembrane proteins whose activity, similarly to MMPs, is dependent on zinc ions. ADAM play an important role in the regulation of cell phenotype by influencing the processes of adhesion, migration, proteolysis and signal transmission [63]. Through their proteolytic and adhesive properties ADAM play an important role in intercellular interactions and in the interactions between cells and ECM. Intercellular interactions occur through changes in the surrounding environment, which is directly related to the release of adhesion molecules, cytokines, growth factors and their receptors from the external surface of the cell membrane. Out of 20 ADAM types present in humans, 12 ADAM types have a strong proteolytic properties [64,65]. Two enzymes of this group, ADAM10 and ADAM17, are crucial for the development of inflammation and the consequential arthropathy. ADAM10 plays a key role in cellular migration and adhesion processes as it mediates the cleavage of transembranous precursors of growth factors and cadherins. ADAM17 is able to release TNF-α and participates in the generation of cytokine receptors, especially IL-6 and TNF-α, thus regulating the inflammatory process [64,66]. ADAM10 expression in healthy cartilage tissue is low, but increases in joint diseases, stimulated by pro-inflammatory cytokines [67,68]. Isozaki et al. [69] showed an increased serum ADAM10 concentration in patients with RA, correlated with the activity of arthropathy. So far, this molecule has not been the subject of studies in children with JIA. 

ADAMTS family are a group of 19 metalloproteinases, summarized in the Table 1, with various functions and substrate spectrum. Due to the fact that the degradation of aggrecan—a major PG of cartilage tissue, is the cause of inflammatory joint diseases, ADAMTS proteins are the subject of research by many scientists. The following 5 members, i.e., ADAMTS1, ADAMTS4, ADAMTS5, ADAMTS8, and ADAMTS9 have been shown to degrade the aggrecan in arthritis [70,71]. ADAMTS4 and ADAMTS5 are considered the main proteases involved in the pathogenesis of inflammatory joint diseases. The suppression of ADAMTS4 and ADAMTS5 expression in cartilage tissue in vitro significantly reduces the processes of aggrecan degradation [72,73,74,75]. ADAMTS proteins, like MMPs and ADAMs, are produced in the form of enzymatically inactive zymogens. A number of factors regulating the production of the ADAMTS proteins are specified e.g., cytokines, i.e., TNF-α, IL-1β, transforming growth factor β (TGF-β), oncostatin M. ADAMTS5, in contrast to ADAMTS4, is constantly synthesized by chondrocytes and fibroblasts of the synovial membrane, thus taking part in physiological processing of the aggrecan [47,73,76,77]. The activity of both enzymes is modulated by the PACE-4 factor. PACE-4 belongs to the group of proprotein convertases, which is released by chondrocytes, but only in pathological conditions of cartilage tissue [78]. Intermolecular interactions may also be a factor regulating enzymatic activity. ADAMTS5 activity is increased by a direct interaction with the membrane PG called syndecan-4 [78,79]. Maintaining the balance between the processes of destruction and synthesis of ECM components is possible due to the presence of endogenous inhibitors of ADAMTS proteins such as α2-macroglobulin, TIMP-3 and fibroblast growth factor 2 (FGF-2- fibroblast growth factor 2). FGF-2 is linked to ECM and released as a result of mechanical injury. It is supposed that FGF-2 prevents aggrecan degradation by inhibiting ADAMTS4 and ADAMTS5 expression induced by IL-1α [78,80]. The enzymatic activity of ADAMTS proteins is also controlled by their internalization and degradation. As an example of these reactions, the enzyme interacts with LRP-1, i.e., protein 1 associated with low-density lipoproteins. The different affinity to LRP-1 for ADAMTS4 and ADAMTS5 results in different half-life periods. Through binding with LRP-1 the main ADAMTS inhibitor’s activity, i.e., TIMP-3 intensifies [41,48].

The studies indicated clearly the dominating role of ADAMTS4 in the destruction of the aggrecan [81]. This is reflected in a significantly higher concentration of the ADAMTS4 in the blood of children with JIA. Treatment with methotrexate, oral corticosteroids and non-steroidal anti-inflammatory drugs results in the normalization of ADAMTS4 level. A similar increase in the level of ADAMTS4 in SF was observed in adults with inflammatory joint diseases, which seems to confirm a significant influence of ADAMTS4 on the metabolism of cartilage ECM components [82].

## 4. Reactive Oxygen and Nitrogen Species 

ROS belong to the group of unstable and highly reactive molecules, among which a special role is played by: Superoxide anion, hydroxyl radical, hydroperoxyl or hydrogen peroxide. The main source of ROS in cells is mitochondrial oxidative phosphorylation processes, as well as interactions with exogenous compounds such as xenobiotics [83]. In homeostasis, ROS play a key role in many physiological processes, i.e., they act as mediators and regulators of cellular metabolism. ROS are also known for their ability to regulate gene expression, apoptosis induction and signal transduction to and within cells [84,85,86,87]. The activity of ROS in physiological conditions is controlled by the antioxidant system. An antioxidant is a substance or compound capable of preventing or reducing the adverse effects of ROS by inhibiting their formation or impact on cellular components and by interrupting free radical chain reactions. The antioxidants include enzymatic systems such as superoxide dismutase, catalase, glutathione peroxidase and glutathione reductase as well as low molecular weight antioxidants e.g., vitamins A, C, E, carotenes, trace elements and glutathione [88,89]. An excessive production of ROS and exhaustion of body antioxidant reservoirs, defined as oxidative stress, leads to oxidation of proteins, lipids, carbohydrates and damage to nucleic acids [90]. ROS inhibits protease inhibitors and stimulates the proteolytic degradation of ECM. ROS are unstable in the collected biological samples so their direct evaluation is limited. The research conducted so far has been mainly based on the determination of parameters resulting from the oxidative activity of ROS, i.e., products of lipid and protein peroxidation [33,91,92].

Prooxidative-antioxidative balance disturbances are among the recognized factors underlying the degradation processes of ECM components in the course of JIA. Development of oxidative stress in children, expressed as increased free radical activity accompanied by the weakening of antioxidative systems, contributes to direct damage to cartilage tissue cells and co-forming components. It is thought that they stimulate chondrocytes and fibroblasts of the synovial membrane to synthesize MMPs, which intensify ECM degradation processes [93,94,95]. The evidence of developing oxidative stress in children with JIA is an increase in total oxidative status, expressed in the concentration of lipid peroxides in patients’ blood [54]. The research by Guney et al. [96] showed that children suffering from JIA have increased levels of malondialdehyde (MDA) in serum, which is a product of lipid oxidation, in comparison to healthy children. The highest levels of MDA were found in blood of patients with systemic arthritis which is characterized by the highest severity. The results by Ramos et al. [97], taking into account the evaluation of carbonyl groups in the blood of children, which are the indicators of protein oxidation, also confirmed the intensification of free radical activity in the course of JIA. Another parameters associated with the development of oxidative stress include myeloperoxidase (MPO). MPO is found in neutrophil granules and monocytes and is released at the inflammation site, contributing to the formation of ROS. Children with JIA have higher plasma MPO levels than healthy. In the group of patients with the arthropathy the highest concentration of MPO is observed in children with polyarticular JIA [98,99].

Apart from the increased activity of free radical processes, weakening of antioxidant systems is a factor leading to oxidative stress development. In children with JIA the decreased activity of enzymes protecting against damaging effects of ROS, i.e., superoxide dismutase, glutathione peroxidase and catalase is observed [96,97,100]. Alternatively, the concentration of thiol groups in the blood serum is measured to reflect the total antioxidant status. The researchers have shown the decrease of the thiol group in children with JIA, especially in the active phase of the disease, which seems to support the role of antioxidants in the etiology of the illness [54,101]. The quantitative evaluation of ceruloplasmine in blood of children with JIA revealed significantly higher concentrations of this antioxidant compared to healthy children [96].

RNS are another, besides ROS, group of highly active agents, which include nitric oxide (NO), nitrosyl cation, nitroxyl anion and peroxynitrite. NO is formed by the enzyme called nitric oxide synthase (NOS). There are three forms of NOS, i.e., endothelial, neuronal and inducible [102]. The activators of the endothelial form include acetylcholine, bradykinin and hydrogen peroxide. NO in the endothelium of blood vessels, is responsible for regulating blood pressure. The neuronal form of the enzyme occurring in central and peripheral nerve cells controls the synthesis of NO, which is responsible for neurotransmission in a mechanism dependent on calcium and calmodulin. Inducible form of NOS is stimulated by the activity of IL-1,-2,-6, TNF-α and interferon γ and is expressed mainly in macrophages, neutrophils and hepatocytes. It is responsible for the formation of large amounts of NO, having cytostatic and toxic effects on target cells [103,104]. It is suggested that the increased endogenous synthesis of NO in inflamed joints is the basis for the development of arthropathy, including JIA. Experiments carried out on animal tissues prove that NO, in response to TNF-α, inhibits the synthesis of PGs, collagen proteins and stimulates the synthesis of MMPs and ADAMTS leading to the destruction of extracellular matrix. NO serves as a mediator in the process of apoptosis and expression of pro-inflammatory cytokines [105,106].

Determination of NO is difficult due to biological instability. Therefore, the final NO metabolism products as nitrite (NO_2_^−^ and nitrate (NO_3_^−^) are measured or the activity of NOS is assessed. Studies by Lipińska et al. [107] indicated that in the group of patients with JIA a significantly higher NO_2_^−^/NO_3_^−^ serum concentration is observed compared to healthy children. Increased NO_2_^−^/NO_3_^−^ concentration were also found in the SF of children with JIA [108]. Patients with diagnosed rheumatoid factor, uveitis and joint erosion demonstrated higher serum NO levels in comparison to patients in the inactive stage of the illness [109]. The evaluation of NOS activity, performed so far in adults with RA, indicates an increased activity of the inducible form of the enzyme [110].

## 5. Anabolic Changes in ECM

A joint cartilage dysfunction in the course of JIA, apart from the excessive proteolysis of ECM components, may also involve disturbed biosynthesis processes of matrix components. A lot of growth factors stimulate PGs synthesis by chondrocytes, induce synoviocyte and mesenchymal stem cells proliferation as well as decrease the proteolitic effects of MMPs [111]. Among the factors that significantly influence the biosynthesis processes of ECM components are TGF-β, bone morphogenetic proteins-2 (BMP-2), platelet-derived growth factor BB (PDGF-BB) and insulin-like growth factor 1 (IGF-1). Changes in the activity of these multifunctional molecules are observed in patients with osteoarticular disorders. During osteolysis by osteoclasts releases large amounts of components from the ECM, mainly TGF-β [112]. This is probably to stimulate the reconstruction of the lysed elements. The TGF-β is a profibrotic group of growth factors which plays a critical role in early embryonic development and postnatal growth and regulates cell proliferation, differentiation, apoptosis and migration [113]. We can distinguish three of them—differentially expressed in tissues or cells—isotypes of TGF-β, i.e., TGF-βs 1, 2 and 3, the activity of which is connected with stimulation of appropriate receptors. Both TGF-β isoforms and TGF-β receptors are extensively expressed in cartilage, bone and synovial tissues. It seems that TGF-β signaling plays a fundamental role in the regulation of chondrocyte homeostasis during cartilage growth and in its remodeling processes. The TGF-β factor strengthens chondrocyte proliferation while inhibiting chondrocyte hypertrophy and maturation. During this process, the expression of genes encoding type II collagen and aggrecan is intensified with the subsequent formation of cartilage tissue. The TGF-β decreases the catabolic activity of IL-1 [111]. The inhibition of TGF-β signaling in chondrocytes leads to chondrocyte terminal differentiation. In synovial fibroblasts, TGF-β signaling is a promoting factor of synovial tissue fibrosis. Fibrosis is described as fibroblast proliferation and accumulation of matrix components, i.e., types I and III collagen. During JIA progression synovial lining hyperplasia and its fibrosis occurs. TGF-β stimulates synovial lining cells to produce cytokines, including pro-inflammatory ones i.e., IL-1 or TNF-α, that can further stimulate articular chondrocytes hypertrophy, accumulating type X collagen instead of type II collagen and aggrecan [114]. The role of the TGF-β in ECM metabolism in children with JIA is not clear and TGF-β1 therapy is not presently a practicable option for use. The research has shown highly increased levels of TGF-β1 in untreated patients with JIA. Clinical improvement is accompanied by a significant decrease in TGF-β compared to the pre-treatment and control group status. A similar tendency of TGF-β changes is observed in adults with inflammatory joint diseases [81,111,113]. The results of Sun et al. [115] indicated an increased serum expression of TGF-β in patients with RA compared to healthy subjects. Superfamily of TGF-β includes BMP-2, which is regarded as a strong factor stimulating the development of the osteoarticular system. BMP-2 also plays significant roles in maintaining adult tissue homeostasis such as the maintenance of joint integrity, the initiation of fracture repair, as well as vascular remodeling [116]. BMP-2 induces an increase in mRNA levels of collagen type II and aggrecan and stimulates PG synthesis in vivo, both in healthy and damaged cartilage. BMP-2 is up-regulated in areas, including mechanically injured cartilage tissue as well as in chondrocytes stimulated with pro-inflammatory cytokines i.e., IL-1 or TNF-α. BMP-2 acts synergistically with TGF-β in joint injuries stimulated by IL-1 influences [117]. So far, BMP-2 has not been studied in children with JIA. Brescla et al. [118] indicated a significant overexpression of BMP-4 in patients with polyarticular JIA, which suggests the role of BMP in ECM metabolism in the course of the JIA. The evidence for the anabolic roles of PDFGs in cartilage turnover is the stimulation of ECM components synthesis in growth plate chondrocytes [111]. The PDGFs’ effect, occurring via receptors (PDGFR-α and PDGFR-β), involves the regulation of fibroblast proliferation, collagen synthesis and angiogenesis. PDGF has a crucial role in the regulation of inflammatory processes. PDGF indirectly affects the expression of membrane receptors for IL-1 which may be reflected in the development of JIA. PDGF, similarly to TGF-β, function as a growth factor for fibroblast-like synoviocytes (FLS), i.e., cells that secrete connective tissue components, including fibronectin, laminin, collagen and PGs [119]. Activated FLS also provide proteinases that degrade the PG components of the joint. PDGF is capable of inhibiting the collagenase synthesis, while increasing IL1β-induced prostaglandin E2 synthesis by FLS. FLS stimulated by PDGF and TGF-β play an important role in the synthesis and degradation of PGs/GAGs. The studies of Rosengren et al. [120] demonstrated that PDGF and TGF-β strongly and selectively enhance the cytokine-induced synthesis and secretion of pro-inflammatory factors by FLS, such as IL-6, IL-8, macrophage inflammatory proteins 1α, and MMP-3. In the studies presented by Winsz et al. [81], an increased level of PDGF-BB in patients with JIA before treatment is observed. The increase of PDGF-BB correlates with the disease activity. The influence of PDGF on ECM metabolism may also occur indirectly through ROS generation. Sundaresan et al. [121] in experimental studies showed that the PDGF and epidermal growth factor can quickly and temporarily increase hydrogen peroxide production by NADPH oxidases. The strongest inducer of ECM component production among the growth factors evaluated so far is IGF-1, which is also the main growth factor for articular cartilage. It plays a key role in cartilage homeostasis, balancing turnover of ECM components, especially PGs. The degree of collagen biosynthesis compared to PGs, does not seem to be equally influenced by IGF-1. Chondrocyte responsiveness to IGF as well as serum IGF levels remains highest in pre- and early puberty and decreases with age [122]. In the course of JIA, IGF-1 shows different patterns of changes in blood compared to TGF-β and PDGF-BB. In the blood of untreated JIA patients, IGF-1 concentration is decreased or its values are comparable to concentration observed in healthy children. Lundell et al. [123] revealed significantly lower IGF-1 levels in patients, mainly in boys, in the early phase of JIA. However, the mechanisms underlying these gender differences are unclear. One hypothesis assumes that adolescence in boys usually starts at a later age than in girls. Probably, increasing estrogen levels in maturing girls indirectly increase the concentration of the growth hormone, which is associated with increase in IGF-1 levels. Wong et al. [124] also indicated low IGF-1 levels in the majority of children with the arthropathy. Guszczyn et al. [125] demonstrated high levels of IGF-1 in the SF collected from patients with diagnosed polyarticular and systemic arthritis in severe stage of the disease. The results indicate that a high level of IGF-1 in SF may be caused by injuries and tissue damage, which results in a local increase in IGF-1 production. The increase in the amount of IGF-1 found in joint exudate may lead to repair of damaged tissues by stimulating cell division [125]. Despite the differences in studies, it is not possible to compare the results due to the determination of factors in two different biological fluids, serum and SF respectively. It is suggested that IGF-1 deficiency may have serious clinical consequences in children with JIA, contributing to local osteoarticular lesions and leading to growth impairment [123,124,125].

## 6. Conclusions

In the course of JIA, there occurred a dysregulation of cartilaginous ECM remodeling, especially aggrecan, manifested by significant changes of the circulating markers of cartilage turnover, including total glycosaminoglycans (assessed by spectrophotometry method) and their particular types such as keratan sulphate (assessed by enzyme-linked immunosorbent assay, ELISA), chondroitin sulphate (assessed by electrophoretic method), hyaluronic acid (assessed by ELISA) as well as chondroitin sulphate 846 epitope (assessed by ELISA) [54,73,81]. Among ECM components, there are also molecules synthesized by chondrocytes, i.e., cartilage oligomeric matrix protein (COMP) and human cartilage glycoprotein 39 (YKL-40) (assessed by ELISA) that can be useful biomarkers of cartilage turnover [126]. These markers can be a useful diagnostic tool to evaluate cartilage condition in JIA patients and could be used to assess treatment towards remission. The ECM components metabolism are related both to the change in the rate of the matrix components degradation processes, which takes place in the presence of excess of ROS (assessed by spectrophotometric method) and proteolytic enzymes—especially MMPs and ADAMTS proteins (assessed by ELISA), as well as to the biosynthesis of matrix compounds [31,32,111]. Treatment that results in clinical stabilization did not contribute to the normalization of markers of cartilage turnover such as chondroitin sulphate [127], keratan sulphate [81], YKL-40 [126] as well as of MMP-3 [43,54], TIMP-1 [60] and TAS concentrations in patients’ blood [54]. JIA therapy with the use of methotrexate, glucocorticoids and the non-steroidal anti-inflammatory drugs, may alleviate symptoms by reducing pain and inflammation, but destruction of connective tissues often continues unabated [43,73]. This indicates the necessity for continuing treatment of patients, despite the resolution of clinical symptoms, and the implementation of a therapy that results in an alignment of the metabolism of cartilage components. Given the destructive potential of ROS and proteinases and their aberrantly high levels of expression in arthritis, reducing or even abolishing these pathologic levels could bring a substantial clinical benefit. Every attempt to inhibit the compounds overproduction in JIA patients would be beneficial, as it may lead to normalization of the ECM components’ metabolism, and thereby to the delay of systemic symptoms of the disease.

## Figures and Tables

**Table 1:** Subtypes of MMPs and ADAMTS and their main substrates [40,46,47,48].

Subtype of MMPs	MMP No.	Main Substrates
collagenases	MMP-1	collagen type I, II, III, V, VII, VIII i X, MMP-2, -9, proteoglycans, fibronectin, laminin.
	MMP-8	collagen type I, II, III, V, VII, VIII, X proteoglycans, fibronectin, ADAMTS-1, proMMP-8.
	MMP-13	collagen type I, II, III, IV, V, IX, X i XI, laminin, proMMP-9, -13.
gelatinases	MMP-2	collagen type I, II, III IV, V, VII, X, elastin, fibronectin, laminin, aggrecan, proMMP-9, -13, IL-1β, TGF-β.
	MMP-9	collagen type IV, V, VII, X, XIV, aggrecan, elastin, fibronectin, laminin, IL-1β, TGF-β, plasminogen.
stromelysins	MMP-3	collagen type III, IV, V, IX, X, XI, elastin, laminin, fibronectin, aggrecan, proMMP-1, -7, -8, -9, -13.
	MMP-10	collagen type I, III, IV, V, elastin, laminin, aggrecan, fibronectin, MMP-1, -8.
	MMP-11	collagen type IV, fibronectin, laminin, aggrecan.
matrilysins	MMP-7	collagen type IV, X, laminin, elastin, fibronectin, proteoglycans, proMMPs, E-cadherin.
	MMP-26	collagen type I, IV, laminin, elastin, fibronectin, proteoglycans, proMMPs, E-cadherin.
membrane type MMP	MMP-14	collagen type I, II, III, fibronectin, vitronectin, aggrecan, perlecan, laminin, proMMP-2, -13.
	MMP-15	collagen type I, II, III aggrecan, perlecan, laminin, fibronectin, proMMP-2.
	MMP-16	collagen type III, proMMP-2.
	MMP-17	proMMP-2, fibronectin, fibrin,
	MMP-24	proMMP-2, -13.
	MMP-25	proMMP-2.
other MMPs	MMP-12	collagen type IV, elastin, fibronectin, vitronectin, laminin.
	MMP-18	collagen type I, II, III.
	MMP-19	collagen type IV.
	MMP-21	collagen type IV.
	MMP-27	collagen type IV.
**Subtypes of ADAMTS**	**ADAMTS No.**	**Main Substrates**
aggrecanases/proteoglycanases	ADAMTS1	aggrecan, versican, syndecan.
	ADAMTS 4	aggrecan, versican, biglycan, brevican.
	ADAMTS 5,	aggrecan, versican, biglycan, brevican.
	ADAMTS 8,	aggrecan.
	ADAMTS 9,	aggrecan, versican.
	ADAMTS 15,	aggrecan, versican.
	ADAMTS 20	versican.
procollagen N-propeptidases	ADAMTS2,	fibrillar procollagens types I-III and V.
	ADAMTS3,	fibrillar procollagen type II, biglycan.
	ADAMTS14	fibrillar procollagen type I.
cartilage oligomeric matrix protein-cleaving enzymes	ADAMTS7,12	cartilage oligomeric matrix protein.
von Willebrand Factor proteinase	ADAMTS13	von Willebrand Factor.
orphan enzymes	ADAMTS6,10,16,17,18,19	unknown.
